# Retraction Note: Diabetes mellitus and risk of incident dementia in APOE ɛ4 carriers: an updated meta-analysis

**DOI:** 10.1186/s12868-026-00995-7

**Published:** 2026-01-13

**Authors:** Ava Rashtchian, Mohammad Hossein Etemadi, Elham Asadi, Sara Binaei, Mina Abbasi, Maedeh Bayani, Erfan Izadi, Sayedeh-Fatemeh Sadat-Madani, Mahdyieh Naziri, Sahar khoshravesh, Mahsa shirani, Mahsa Asadi Anar, Niloofar Deravi

**Affiliations:** 1 Student Research Committee, School of Medicine, Shahid Beheshti University of Medical Sciences, SBUMS, Arabi Ave, Daneshjoo Blvd, Velenjak, Tehran 19839-63113 Iran; 2 Students Research Committee, School of Medicine, Isfahan University of Medical Sciences, Isfahan, Iran; 3 Endocrinology and Metabolism Research Center, Hormozgan University of Medical Sciences, Bandar Abbas, Iran; 4 Student Research Committee, School of Medicine, Mashhad University of Medical Sciences, Mashhad, Iran; 5 Student Research Committee, Shahid Beheshti University of Medical Sciences, Tehran, Iran; 6 Student Research Committee, School of Medicine, Shahroud University of Medical Sciences, Shahroud, Iran; 7 Medical Doctor, School of Medicine, Isfahan University of Medical Sciences, Isfahan, Iran; 8 Student Research Committee, School of Health, Iran University of Medical Science, Tehran, Iran; 9 Shahid Beheshti University of Medical Sciences, Tehran, Iran

**Retraction Note: BMC Neuroscience (2024) 25:28**

10.1186/s12868-024-00878-9

The Editors have retracted this article. After publication, significant concerns were raised regarding the methodology used for article search and selection in the meta-analysis, as well as inconsistencies in the reporting of results. In response to these concerns, the authors proposed revisions; however, the changes required to address these issues substantially altered the originally reported data and conclusions. As a result, the integrity of the published version can no longer be assured.

Ava Rashtchian and Mahsa Asadi Anar agree with this retraction. The other authors have not responded to any correspondence from the editor or publisher about this retraction.

